# Multipotent mesenchymal stromal cells from patients with newly diagnosed type 1 diabetes mellitus exhibit preserved in vitro and in vivo immunomodulatory properties

**DOI:** 10.1186/s13287-015-0261-4

**Published:** 2016-01-18

**Authors:** Juliana Navarro Ueda Yaochite, Kalil Willian Alves de Lima, Carolina Caliari-Oliveira, Patricia Vianna Bonini Palma, Carlos Eduardo Barra Couri, Belinda Pinto Simões, Dimas Tadeu Covas, Júlio César Voltarelli, Maria Carolina Oliveira, Eduardo Antônio Donadi, Kelen Cristina Ribeiro Malmegrim

**Affiliations:** Department of Biochemistry and Immunology, Basic and Applied Immunology Program, School of Medicine of Ribeirão Preto, University of São Paulo, Av. Bandeirantes, 3900, Monte Alegre, 14049-900 Ribeirão Preto, São Paulo Brazil; Department of Clinical and Toxicological Analysis, Federal University of Ceará, Alexandre Baraúna 949, Rodolfo Teófilo, 60430-160 Fortaleza, Ceará Brazil; Regional Blood Center of Ribeirão Preto, University of São Paulo, Tenente Catão Roxo, 2501, Monte Alegre, 14051-140 Ribeirão Preto, São Paulo Brazil; Department of Clinical Medicine, School of Medicine of Ribeirão Preto, University of São Paulo, Tenente Catão Roxo, 2501, Monte Alegre, 14051-140 Ribeirão Preto, São Paulo Brazil; Department of Clinical, Toxicological and Bromatological Analysis, School of Pharmaceutical Sciences of Ribeirão Preto, University of São Paulo, Av. do Café, Monte Alegre, 14040-903 Ribeirão Preto, São Paulo Brazil

**Keywords:** Mesenchymal stromal cells, Type 1 diabetes mellitus, Immunomodulation, Cell transplantation, Streptozotocin-induced diabetes

## Abstract

**Background:**

Type 1 diabetes mellitus (T1D) is characterized by autoimmune responses resulting in destruction of insulin-producing pancreatic beta cells. Multipotent mesenchymal stromal cells (MSCs) exhibit immunomodulatory potential, migratory capacity to injured areas and may contribute to tissue regeneration by the secretion of bioactive factors. Therefore, MSCs are considered as a promising approach to treat patients with different autoimmune diseases (AID), including T1D patients. Phenotypical and functional alterations have been reported in MSCs derived from patients with different AID. However, little is known about the properties of MSCs derived from patients with T1D. Since autoimmunity and the diabetic microenvironment may affect the biology of MSCs, it becomes important to investigate whether these cells are suitable for autologous transplantation. Thus, the aim of the present study was to evaluate the in vitro properties and the in vivo therapeutic efficacy of MSCs isolated from bone marrow of newly diagnosed T1D patients (T1D-MSCs) and to compare them with MSCs from healthy individuals (C-MSCs).

**Methods:**

T1D-MSCs and C-MSCs were isolated and cultured until third passage. Then, morphology, cell diameter, expression of surface markers, differentiation potential, global microarray analyses and immunosuppressive capacity were in vitro analyzed. T1D-MSCs and C-MSCs therapeutic potential were evaluated using a murine experimental model of streptozotocin (STZ)-induced diabetes.

**Results:**

T1D-MSCs and C-MSCs presented similar morphology, immunophenotype, differentiation potential, gene expression of immunomodulatory molecules and in vitro immunosuppressive capacity. When administered into diabetic mice, both T1D-MSCs and C-MSCs were able to reverse hyperglycemia, improve beta cell function and modulate pancreatic cytokine levels.

**Conclusions:**

Thus, bone marrow MSCs isolated from T1D patients recently after diagnosis are not phenotypically or functionally impaired by harmful inflammatory and metabolic diabetic conditions. Our results provide support for the use of autologous MSCs for treatment of newly diagnosed T1D patients.

**Electronic supplementary material:**

The online version of this article (doi:10.1186/s13287-015-0261-4) contains supplementary material, which is available to authorized users.

## Background

Type 1 diabetes mellitus (T1D) is a chronic disease characterized by an autoimmune response in which cellular immunity plays a pivotal role in the selective destruction of insulin-producing pancreatic beta (β) cells, thus leading to metabolic dysfunction. While insulin replacement stands as the main therapeutic approach for T1D patients, it is insufficient to prevent long-term complications such as vascular dysfunction, retinopathy, and kidney failure [1]. Possible alternative treatments, such as human pancreas and islet transplantation, are limited by lack of sufficient donors, high costs, and need for chronic post-transplant immunosuppression [2]. In addition, the immune regulation and preservation of the β-cell mass have been attempted by the administration of immunosuppressive agents such as prednisone, azathioprine, and cyclosporine in several clinical trials. However, adverse effects of such drugs and the need for continuous treatment have limited the application of these therapies [3]. These difficulties have prompted research into the development of alternative and innovative methods to treat T1D patients.

In this sense, adult stem cell transplantation represents a promising possibility that must be explored. High-dose immunosuppression followed by autologous hematopoietic stem cell transplantation was shown to increase C-peptide levels with reduction or even suspension of insulin use in the majority of newly diagnosed T1D patients [4]. In addition, multipotent mesenchymal stromal cells (MSCs) have also attracted great attention as a powerful tool for T1D treatment because of their regenerative and immunomodulatory properties.

MSCs are multipotent mesenchymal precursors found in several tissues. Considering the possible perivascular origin of MSCs, it has been suggested that MSCs may be present in any vascularized tissue throughout the whole body [5]. MSCs express different nonspecific surface molecules (including CD90, CD73, CD105, CD29, CD44, and CD166), but do not express endothelial or hematopoietic markers (CD31, CD45, CD43, CD14, CD11b), major histocompatibility complex (MHC) class II molecule, and costimulatory proteins (CD80, CD86, CD40) [6, 7]. The first described MSC function was to provide cytokines and growth factors to support the hematopoietic process. Moreover, these cells have the capacity to differentiate in vitro into cell types from connective tissue such as adipocytes, chondroblasts, and osteoblasts [8]. In addition, MSCs migrate to injured tissues and may promote regeneration by the secretion of several bioactive factors [9].

MSCs have been shown to have immumodulatory and immunosuppressive properties, both in vitro and in vivo [10]. MSCs modulate the function of T and B lymphocytes [11, 12], dendritic cells [13], natural killer cells [14], and regulatory T (Treg) cells [15]. Cell-to-cell contact and the production of immumodulatory soluble factors such as transforming growth factor beta (TGF-β), indoleamine 2,3-dioxygenase (IDO), prostaglandin E_2_, interleukin (IL)-10, and hepatic growth factor (HGF) are involved in these processes [16]. On the other hand, MSCs have their regulatory functions activated (“licensed”) by inflammatory cytokines such as tumor necrosis factor alpha (TNFα), interferon gamma (IFNγ), and IL-1β, as well as by Toll-like receptor (TLR) signaling [17].

In the last decades, MSCs have been the focus of cell-based therapy research, especially for treatment of inflammatory and autoimmune diseases (AID) [18]. MSCs have been extensively used to treat chemically-induced and spontaneous experimental T1D models. Administration of MSCs in diabetic mice/rats delayed the onset of disease, significantly decreased blood glucose levels, increased endogenous insulin production, modulated the expression of cytokines, reduced the pancreatic inflammatory process, and induced the expansion of Treg cells [19–21]. These promising results have stimulated ongoing worldwide clinical trials to test safety and therapeutic potential of MSCs in T1D patients [22]. Noteworthy, most of these trials use allogeneic MSCs from healthy individuals instead of autologous cells. Generally, autologous rather than allogeneic cells are preferred in the transplantation setting, avoiding the risks of immune rejection or transfer of donor-derived infections and other diseases [23]. In this perspective, a fundamental question that must be addressed is whether MSCs from patients with AID preserve their functional properties or may be somehow compromised [24, 25].

Phenotypical and functional characteristics of MSCs derived from patients with different AID have been investigated in the past years [26]. MSCs from systemic sclerosis (SS) patients and from healthy donors exhibited similar proliferation rates, differentiation potential, and in vitro T-lymphocyte inhibition capacity [27]. Conversely, MSCs isolated from patients with rheumatoid arthritis (RA) [28], multiple sclerosis (MS) [29], and immune trombocytopenic purpura (ITP) [30] showed defects in critical cell functions. These alterations might be due to disease-related cellular, molecular, and/or biochemical changes in the bone marrow microenvironment.

In T1D setting, the autoimmune process and metabolic alterations could affect MSCs properties. To date, to our knowledge there are no existing data regarding biological and immunological profiles of MSCs isolated from newly diagnosed T1D patients. Whether these cells are phenotypically and/or functionally abnormal is thus an important question to be addressed in the context of autologous transplantation. The purpose of the present study is therefore to evaluate the in vitro properties and the in vivo therapeutic efficacy of MSCs isolated from newly diagnosed T1D patients in experimental diabetes.

## Methods

### Subjects

Bone marrow samples were obtained from the iliac crest of newly diagnosed T1D patients and healthy donors, after informed consent. Patients (all males; 23.2 ± 2.9 years; 273.83 ± 31.09 mg/dl fasting blood glucose; 9.5 ± 0.7 % HbA1c levels; 19.3 ± 2.0 mean body mass index) enrolled in this study were diagnosed with T1D in the previous 6 weeks, confirmed by positive serum levels of anti-glutamic acid decarboxylase (anti-GAD) antibodies (22.66 ± 15.04 U/ml) and without previous episodes of diabetic ketoacidosis. All patients presented symptoms of hyperglycemia (polyuria, polydipsia, and weight loss) at diagnosis. Healthy subjects (all males; 33.1 ± 4.9 years; fasting blood glucose <100 mg/dl) were recruited among voluntary bone marrow donors. All human procedures were approved by the Ethics Committee of the University Hospital (Ribeirão Preto Medical School, Ribeirão Preto, Brazil), at the University of São Paulo, Ribeirão Preto, Brazil (# 10095/02).

### MSC isolation, culture, and characterization

Bone marrow aspirate samples were collected in presence of ethylenediamine tetraacetic acid (EDTA), and mononuclear cells were separated using Ficoll-Hypaque (Amersham-Pharmacia, Uppsala, Sweden) gradient density. Subsequently, the mononuclear cell layer was harvested, washed, centrifuged, and resuspended in alpha minimum essential medium (αMEM; Gibco Life Technologies, Grand Island, NY, USA) supplemented with 15 % fetal bovine serum (FBS; Thermo Scientific, Rockford, IL, USA), 100 μg/ml penicillin (Gibco), 100 μg/ml streptomycin (Gibco), and 2 mM l‐glutamine (Gibco). The cells were then seeded in 75 cm^2^ flasks and incubated at 37 °C with 5 % CO_2_. After 7 days, nonadherent cells were removed and fresh medium was added twice a week. When layers were confluent, the cells were detached using trypsin (Gibco) and subcultured until third passage. The methods used for analysis of morphology, immunophenotypic profile, and adipocyte differentiation potential of bone marrow-isolated MSCs are described in Additional file 1.

### Microarray analysis

Total RNA was isolated from MSCs from bone marrow of healthy individuals (C-MSCs; *n* = 4) and MSCs from bone marrow of newly diagnosed T1D patients (T1D-MSCs; *n* = 4) using Trizol (Invitrogen LifeTechnologies, Carlsbad, CA, USA), according to the manufacturer’s instructions, purified with the RNeasy mini Kit (Qiagen, Valencia, CA, USA), and analyzed by spectrophotometry at 260 and 280 nm (NanoDrop, ND‐1000 UV‐VIS; Thermo Fisher Scientific, Walthman, MA, USA). Global gene expression analyses were performed by the One-color Microarray-Based Gene Expression Analysis Protocol system (Agilent Technologies, Santa Clara, CA, USA) on glass slides with four microarrays of 44,000 probes each (4 × 44 k; Agilent Technologies). The preprocess and statistical microarray analyses were performed using algorithms available from the R platform the Linear Models for Microarray Data (LIMMA, R Foundation, Vienna, Austria) package. The heatmaps were generated by the HeatMapViewer module from GenePattern 2.0 software (Broad Institute, Cambridge, MA, USA). Genes exhibiting *P* <0.05 and differences in expression of at least 2.0-fold (up or down) were considered statistically significant. Microarray data were deposited in the public database ArrayExpress (http://www.ebi.ac.uk/arrayexpress), access code E-MTAB-2976.

### Lymphocyte proliferation assay

To test the inhibitory effects of T1D-MSCs and C-MSCs on allogeneic lymphocyte proliferation, the carboxyfluorescein diacetate succinimidyl ester (CFSE; Invitrogen LifeTechnologies) dilution method was used. Peripheral blood mononuclear cells (PBMCs) obtained from healthy donors were separated by Ficoll-Hypaque density gradient (Amersham-Pharmacia), labeled with CFSE (10 μM, for 10 minutes at 37 °C), and resuspended in RPMI 1640 medium (Gibco) supplemented with 5 % human serum albumin (Vialebex® 200 mg/ml; LFB, Rio de Janeiro, Brazil). CFSE-labeled PBMCs were added to the wells containing previously adhered patient or control MSCs, in six different ratios (MSCs:PBMCs = 1:2, 1:5, 1:10, 1:20, 1:50, and 1:100) in the presence of 0.5 μg/ml phytohemagglutinin (PHA; Sigma‐Aldrich, St. Louis, MO, USA). The cocultures were incubated for 5 days at 37 °C with 5 % CO_2._ Subsequently, PBMCs were harvested, stained with anti‐CD3 antibody (BD, San Jose, CA, USA) and the dilution of CFSE in CD3^+^ T cells was analyzed by flow cytometry using FACSCalibur™ (BD) equipment.

### In vivo analysis: experimental design

In vivo experiments were designed according to the protocol represented in Additional file 2: Figure S1.

### Induction of experimental diabetes

C57BL/6 male mice 10 weeks of age were intraperitoneally injected with 40 mg/kg streptozotocin (STZ; Sigma-Aldrich) for 5 consecutive days. STZ was diluted in sodium citrate buffer, pH 4.5. Blood samples were taken from the tail vein of nonfasting mice, and glucose levels determined with a glucometer system Accu-Chek Active (Roche Diagnostics, Abbott Park, IL, USA). Mice were considered diabetic when glycemia exceeded 250 mg/dl in two consecutive determinations. All animal procedures were approved by the Ethics Committee for Animal Research of the Ribeirão Preto Medical School (# 157/2010; # 021/2013-01).

### Intrasplenic transplantation of MSCs

Single doses of 1 × 10^6^ T1D-MSCs or C-MSCs were injected into the spleens of diabetic mice (*n* = 9/group) 20 days after the last dose of STZ. The control group received intrasplenic injections of phosphate-buffered saline (PBS; *n* = 6).

For intrasplenic injections of MSCs or PBS, mice were anaesthetized with a mixture of ketamine (Ketamina-Agener União, São Paulo, Brazil) and xylazine (Dopaser-Hertape Calier, Minas Gerais, Brazil). The spleens were exposed after skin and peritoneum incisions and received single MSCs or PBS microinjection (70 μl of final volume). Bleeding was controlled using cotton swabs and local application of fibrin sealant. Incisions were sutured with a 5–0 nylon monofilament (Bioline Fios Cirúrgicos Ltda, Goiás, Brazil). Intraperitoneal injections of tramadol hydrochloride (30 mg/kg, Tramal; Medley, Campinas, Brazil) were used as pain relief every 12 hours for 3 consecutive days.

Nonfasting glucose was monitored every 5 days using the glucometer system Accu-Chek Active (Roche Diagnostics). Mice were sacrificed 35 days after MSC or PBS injection and the pancreas, spleen, pancreatic lymph nodes, and blood samples were collected (Additional file 2: Figure S1).

### Glucose tolerance test

Peripheral response to glucose was evaluated by intraperitoneal glucose tolerance test (GTT) 30 days after C-MSC or T1D-MSC transplantation. Glucose solution (1.5 mg/g body weight) was intraperitoneally administrated in 10-hour fasting mice, and blood glucose levels were determined before and 15, 30, 60, and 180 minutes after glucose administration.

### Histology and immunohistochemistry analysis

For histologic analysis, pancreata were removed, fixated in 10 % neutral buffered formalin, and embedded in paraffin and the sections (5 μm) were stained with hematoxylin and eosin (H & E). Immunohistochemistry reactions were performed on formalin-fixed or frozen Tissue-Tek O.C.T (Sakura Finetek, Zoeterwoude, the Netherlands) tissue sections. First, sections were incubated with Peroxidase-Blocking Reagent (DAKO Cytomation, Fort Collins, CO, USA) to block endogenous peroxidase. The slides were then incubated with a blocking solution containing PBS/bovine albumin serum 1 % (Sigma)/Triton X-100 (BioRad, Richmond, CA, USA) to prevent unspecific staining. Next, rabbit monoclonal anti-mouse insulin antibody (Santa Cruz Biotechnology, Santa Cruz, CA, USA) or rabbit anti-mouse Ki-67 antibody (Abcam, Cambridge, UK) were applied to the sections, followed by incubation with LSAB™ + Kit/HRP (DAKO Cytomation). The slides were stained with diaminobenzidine according to the manufacturer’s instructions (DAKO Cytomation). Finally, the sections were counterstained with Harris hematoxylin and analyzed under light microscopy.

### Isolation of cells from spleens and pancreatic lymph nodes

Each spleen was mashed and the resulting cell suspension collected. Next, erythrocytes were lysed using Tris 0.17 M + NH_4_Cl 0.16 M buffer. Tubes containing the splenocytes received RPMI 1640 medium (Gibco) supplemented with 10 % FBS, 2 mmol/l l-glutamine (Gibco), and 100 U/ml penicillin/streptomycin (Gibco) and were centrifuged at 300 × *g* for 10 minutes at 4 °C. The supernatants were then discarded and pellets resuspended in RPMI 1640 medium (Gibco). Pancreatic draining lymph nodes (PLN) were collected and mashed through a cell strainer into a Petri dish containing RPMI 1640 medium (Gibco). The cell suspension was then collected and centrifuged at 300 × *g* for 10 minutes at 4 °C.

### Flow cytometry analysis of CD4^+^CD25^+^Foxp3^+^ Treg cell population

First, the cell suspension (splenocytes or PLNs) was incubated with 100 μl rabbit normal serum 5 % for 30 minutes to block nonspecific binding. Next, fluorochrome-conjugated primary antibodies against CD4 and CD25 antigens and their control isotypes (BD) were added and incubated for 30 minutes at room temperature in the dark. All monoclonal antibodies were used at concentrations recommended by the manufacturer (BD). After extracellular antigen staining, cells were incubated with FACS Lysing solution (BD) for 10 minutes in the dark. They were then washed and resuspended in FACS permeabilizing solution (BD) for 10 minutes. Next, the expression of the transcription factor Foxp3 was assessed by incubating with PE-conjugated anti-mouse Foxp3 monoclonal antibody (BD). Cell suspension was washed, resuspended, and analyzed using a FACSCalibur™ flow cytometer (BD). Data were obtained for 100,000 events/sample (spleen) or 50,000 events/sample (PLN) using CellQuest Pro software (BD).

### Quantification of cytokine levels in serum and in pancreatic tissue

Pieces of pancreas were removed, weighed, and placed in a tube containing 700 μl Complete Protease Inhibitor Cocktail (Roche Diagnostics). Pancreatic tissue was homogenized using a Polytron homogenizer (Kinematica, Luzern, Switzerland) and IL-2, IL-6, IFNγ, TNFα, IL-17, IL-4, and IL-10 levels were detected by the cytometric bead array (CBA) (Th1/Th2/Th17 kit; BD) method, according to the manufacturer’s instructions. The concentration of TGF-β in pancreatic tissue was determined using Human/Mouse TGF-β1 ELISA Ready-Set-Go kit (eBioscience, San Diego, CA, USA). Serum cytokine levels were also determined by the CBA method.

### Quantification of serum insulin

Blood samples of nonfasting mice were collected 35 days after MSC/PBS administration. The serum insulin concentration was determined using the Mouse Ultrasensitive Insulin ELISA kit (Alpco Diagnostics, Salem, MA, USA), according to the manufacturer’s instructions.

### Statistical analysis

Data are presented as mean ± standard deviation (SD). Statistical comparisons included unpaired/paired *t* tests or one-way analysis of variance with Tukey’s post test. *P* <0.05 was considered significant.

## Results

### T1D-MSCs exhibit morphology, immunophenotypic profile, and adipocyte differentiation capacity similar to MSCs from healthy counterparts

To characterize T1D-MSCs, we evaluated the cell morphology, cell diameter, expression of surface markers, and in vitro differentiation potential, and compared results with those from their healthy counterparts.

**Fig. 1:**
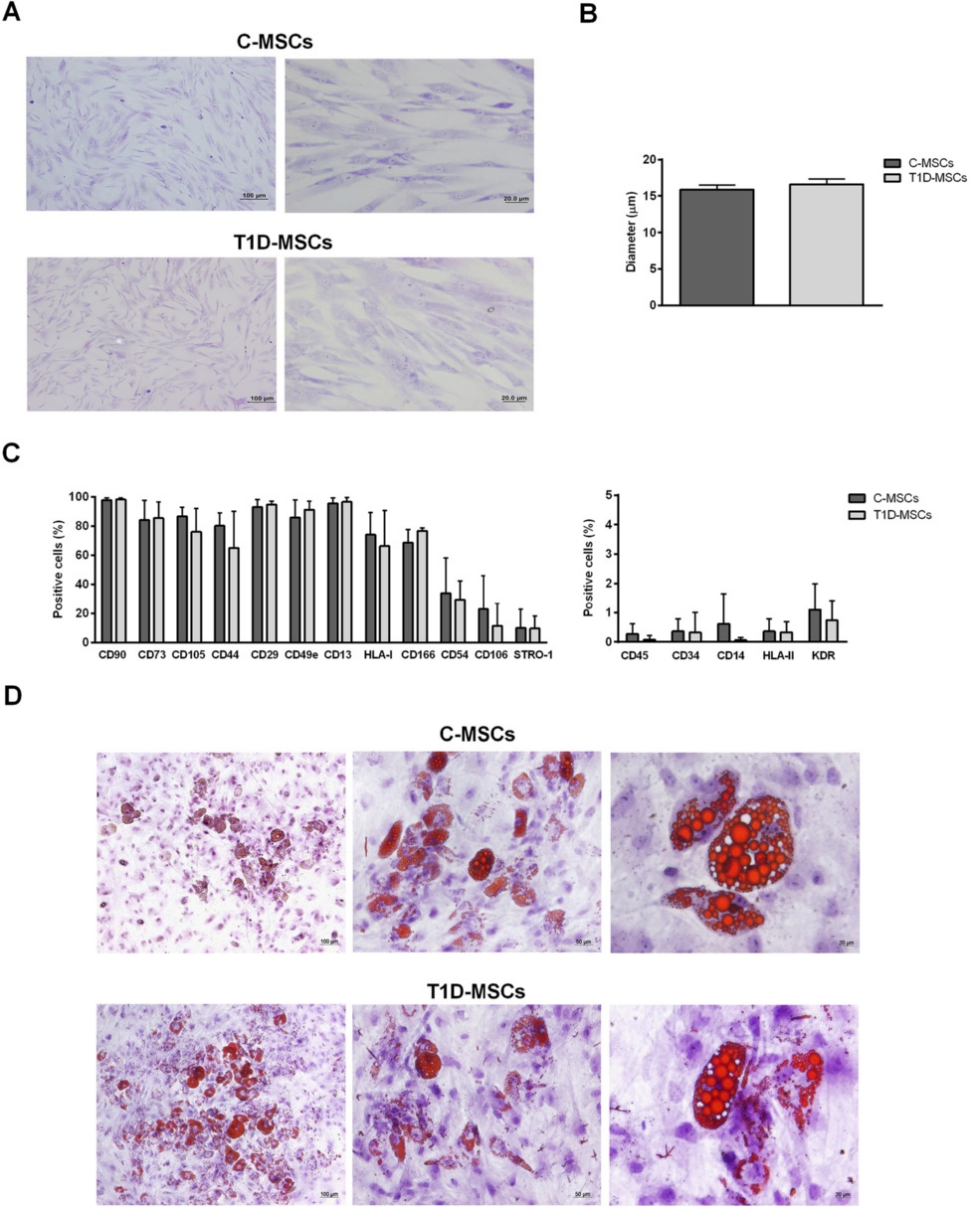
Characterization of T1D-MSCs isolated. **a** Morphological characterization. At the third passage, C-MSCs (*upper panel*) and T1D-MSCs (*lower panel*) showed homogeneous spindle-shaped fibroblast-like growth (Leishman staining, 100× and 400× magnification, respectively). **b** The diameter of C-MSCs (*n* = 5) and T1D-MSCs (*n* = 5) in suspension was determined by ViCell XR equipment (2000 cells/sample). **c** Expression of surface immunophenotypic markers. Graphs display the phenotype of MSCs in culture at the third passage. MSCs from T1D patients were phenotypically similar to those from healthy donors. Bars represent mean ± SD. **d** In vitro adipocyte differentiation. C-MSCs (*upper panel*) and T1D-MSCs (*lower panel*) were able to differentiate towards adipogenic lineage. The presence of lipid vacuoles in the cytoplasm was identified by Sudan II-Scarlet staining (100×, 200×, and 400× magnification, respectively). *C-MSCs* mesenchymal stromal cells from bone marrow of healthy individuals, *T1D-MSCs* mesenchymal stromal cells from bone marrow of newly diagnosed T1D patients

MSCs isolated from bone marrows of healthy donors (C-MSCs) and newly diagnosed T1D patients (T1D-MSCs) appeared as typical monolayers of spindle-shaped fibroblast-like cells, with ability to adhere to plastic during in vitro expansion. At the third passage, T1D-MSCs in culture were morphologically similar to C-MSCs (Fig. 1a). The mean cell size (diameter) of T1D-MSCs in suspension (15.32 ± 0.56 μm) was also similar to that of C-MSCs (15.35 ± 0.71 μm; Fig. 1b).

T1D-MSC and C-MSC samples presented typical MSC phenotype and similar percentages of CD73, CD90, CD105, CD29, CD13, CD44, CD49e, CD54, HLA-I, CD166, CD106, STRO-1, CD45, CD14, CD34, HLA-II, CD51/61, and KDR positive cells (Fig. 1c).

To evaluate the in vitro adipogenic differentiation potential, T1D-MSCs and C-MSCs were cultured for 21 days with specific medium to induce differentiation into adipocytes. T1D-MSCs and C-MSCs were both able to differentiate towards the adipogenic lineage, and cytoplasmic lipid vesicles were detected by Sudan II-Scarlet staining (Fig. 1d). Adipocyte size was determined using morphometric analysis and no differences were observed comparing both MSC sources (C-MSCs 1.19 ± 0.54 × 10^3^ μm^2^ versus T1D-MSCs 1.07 ± 0.62 × 10^3^ μm^2^, *P* >0.05; data not shown)*.*

### Gene expression of immunomodulatory molecules in T1D-MSCs is similar to C-MSCs

**Fig. 2:**
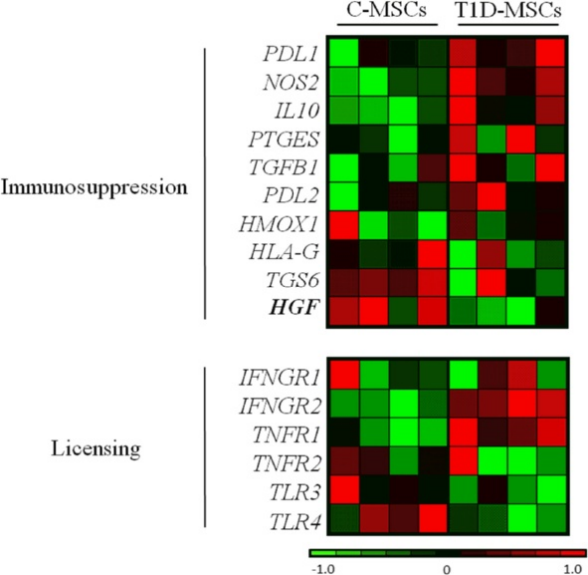
Heatmap of relative differential gene expression of immunosuppressive and licensing-related molecules in C-MSCs and T1D-MSCs. Global gene expression analysis was performed in third-passage C-MSCs (*n* = 4, *left panel*) and T1D-MSCs (*n* = 4, *right panel*). Downregulated genes are shown in *green*. Upregulated genes are presented in *red*. Differentially expressed genes are shown in *bold* (*P* <0.05, fold-change >2). Microarray data were deposited in the public database ArrayExpress (http://www.ebi.ac.uk/arrayexpress), access code E-MTAB-2976. *C-MSCs* mesenchymal stromal cells from bone marrow of healthy individuals, *T1D-MSCs* mesenchymal stromal cells from bone marrow of newly diagnosed T1D patients

To evaluate the differential expression of genes that code immunomodulatory molecules and factors involved in MSC licensing, we performed global microarray analyses of T1D-MSCs and C-MSCs. No significant differences (*P* >0.05, fold-change ≥2) were observed in the expression of *PDL1*, *NOS2*, *IL10*, *PTGES*, *TGFB1*, *PDL2*, *HLAG*, and *TGS6* genes in T1D-MSCs compared with C-MSCs. However, the *HGF* gene was significantly downregulated in T1D-MSCs. The expression of licensing-related genes (*IFNGR1*, *IFNGR2*, *TNFR1*, *TNFR2*, *TLR3*, *TLR4*) was also similar in T1D-MSCs compared with C-MSCs (Fig. 2).

### T1D-MSCs exhibit preserved in vitro immunosuppressive potential

**Fig. 3:**
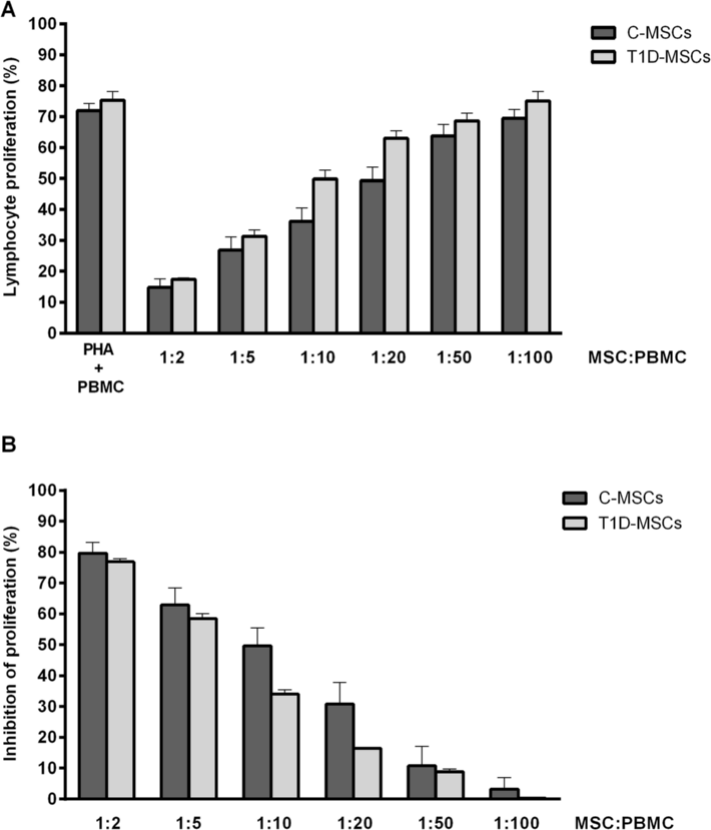
T1D-MSCs efficiently inhibit in vitro T-cell proliferation. In coculture assays, different concentrations of phytohemagglutinin (*PHA*)-stimulated allogeneic peripheral blood mononuclear cells (*PBMCs*) labeled with CFSE were cultured in the presence of C-MSCs (*n* = 6) or T1D-MSCs (*n* = 3). The percentage of CD3^+^ T-lymphocyte proliferation was determined by flow cytometry. **a** Percentages of lymphocyte proliferation. **b** Percentages of lymphocyte proliferation inhibition. Data expressed as mean ± SD. *C-MSCs* mesenchymal stromal cells from bone marrow of healthy individuals, *MSC* multipotent mesenchymal stromal cell, *T1D-MSCs* mesenchymal stromal cells from bone marrow of newly diagnosed T1D patients

Once T1D-MSCs showed no significant alterations in the expression of immunomodulatory genes, we comparatively assessed their immunosuppressive capacity in cocultures with activated PBMCs. T1D-MSCs and C-MSCs were able to efficiently suppress the proliferation of CD3^+^ cells in a dose‐dependent manner, in the ratios (MSCs/PBMCs) of 1/2, 1/5, 1/10, and 1/20. Comparing the suppressive capacity of T1D-MSCs with C-MSCs, no significant differences were observed in all evaluated concentrations (Fig. 3a, b).

### Administration of T1D-MSCs promotes glycemic control and improves peripheral response to glucose in STZ-induced diabetic mice

Since our in vitro analyses demonstrated that T1D-MSCs presented morphological, transcriptional, and in vitro functional characteristics similar to C-MSCs, we tested their therapeutic potential using a murine experimental model of STZ-induced diabetes. Single doses of 1 × 10^6^ cells (T1D-MSCs or C-MSCs) were directly injected into the spleen of diabetic mice, while mice from the diabetic control group received intrasplenic injection of PBS (Control-PBS).

**Fig. 4:**
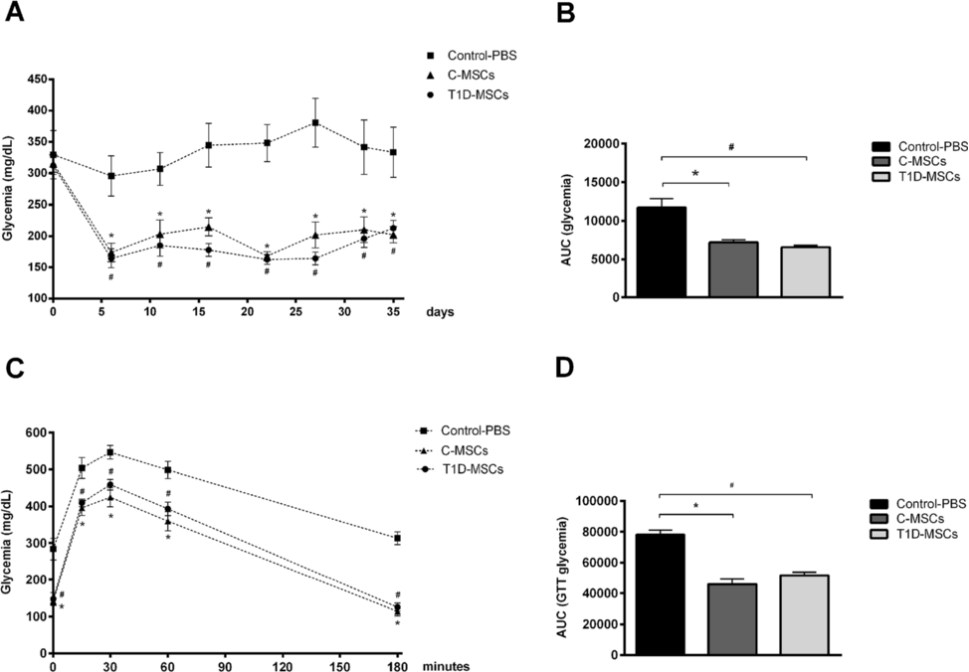
Intrasplenic injection of T1D-MSCs promotes reversion of hyperglycemia and improvement of peripheral response to glucose in STZ-induced diabetic mice. T1D-MSCs or C-MSCs (1 × 10^6^) were administered by intrasplenic injection in diabetic mice 20 days after diabetes induction. **a** Blood glucose levels (mg/dl) were frequently measured in nonfasting mice for 35 days. A reduction in blood glucose levels was observed in 67 % (6/9) of diabetic MSC-treated mice mainly on day 6 post transplantation (only the glycemic levels of responder mice/*n* = 6 are shown). The control group was treated with intrasplenic injection of PBS (Control-PBS, *n* = 6). **b** Area under the curve of glycemia (*AUC*) from day 0 to day 35. The AUC was determined for each animal, and the mean ± SD of each group is shown. **c** Glucose tolerance tests (*GTT*) were performed in 10-hour fasted mice, 30 days after T1D-MSC, C-MSC, or PBS intrasplenic administration. Glucose (1.5 mg/g) was intraperitoneally administered and blood glucose levels (mg/dl) were determined 0, 15, 30, 60, and 180 minutes after administration. **d** AUC during GTT was determined for each animal and the means ± SD of each group is shown. *P <0.05 (Control-PBS × C-MSCs); ^#^ *P* <0.05 (Control-PBS × T1D-MSCs). *C-MSCs* mesenchymal stromal cells from bone marrow of healthy individuals, *PBS* phosphate-buffered saline, *T1D-MSCs* mesenchymal stromal cells from bone marrow of newly diagnosed T1D patients

The administration of T1D-MSCs or C-MSCs equally reversed hyperglycemia in 67 % (6/9) of mice (responder mice). Blood glucose levels of T1D-MSC-treated or C-MSC-treated responder mice were significantly lower compared with those from the control group during follow-up (Fig. 4a). The area under the glycemia curve (AUC) of the C-MSC-treated responder group (7183.11 ± 839.11) was similar to the AUC of the T1D-MSC-treated responder group (6568.33 ± 604.96). On the other hand, AUCs were significantly lower in the cell-treated groups than in the control group (11,728.17 ± 2805.10, *P* = 0.0002; Fig. 4b). The fasting glycemia of the C-MSC-treated (141.8 ± 9.08 mg/dl) and the T1D-MSC-treated (147.1 ± 17.53 mg/dl) responder groups were lower than that from the control group (283.8 ± 29.50 mg/dl, *P* < 0.0001; data not shown). Noteworthy, 100 % of T1D-MSC-treated and C-MSC-treated mice exhibited fasting glucose levels lower than 250 mg/dl.

Thirty days after treatment with T1D-MSCs or C-MSCs, responder animals had improved response to exogenous glucose compared with the control group (Fig. 4c). Significant differences were observed when comparing the GTT AUC of T1D-MSC-treated (51,740.33 ± 5005.53) and C-MSC-treated (45,880.17 ± 8219.11) responder mice with that from the control group (78,152.67 ± 6979.88, *P* <0.0001; Fig. 4d).

### T1D-MSC treatment decreases pancreatic inflammation and improves pancreatic β-cell function

**Fig. 5:**
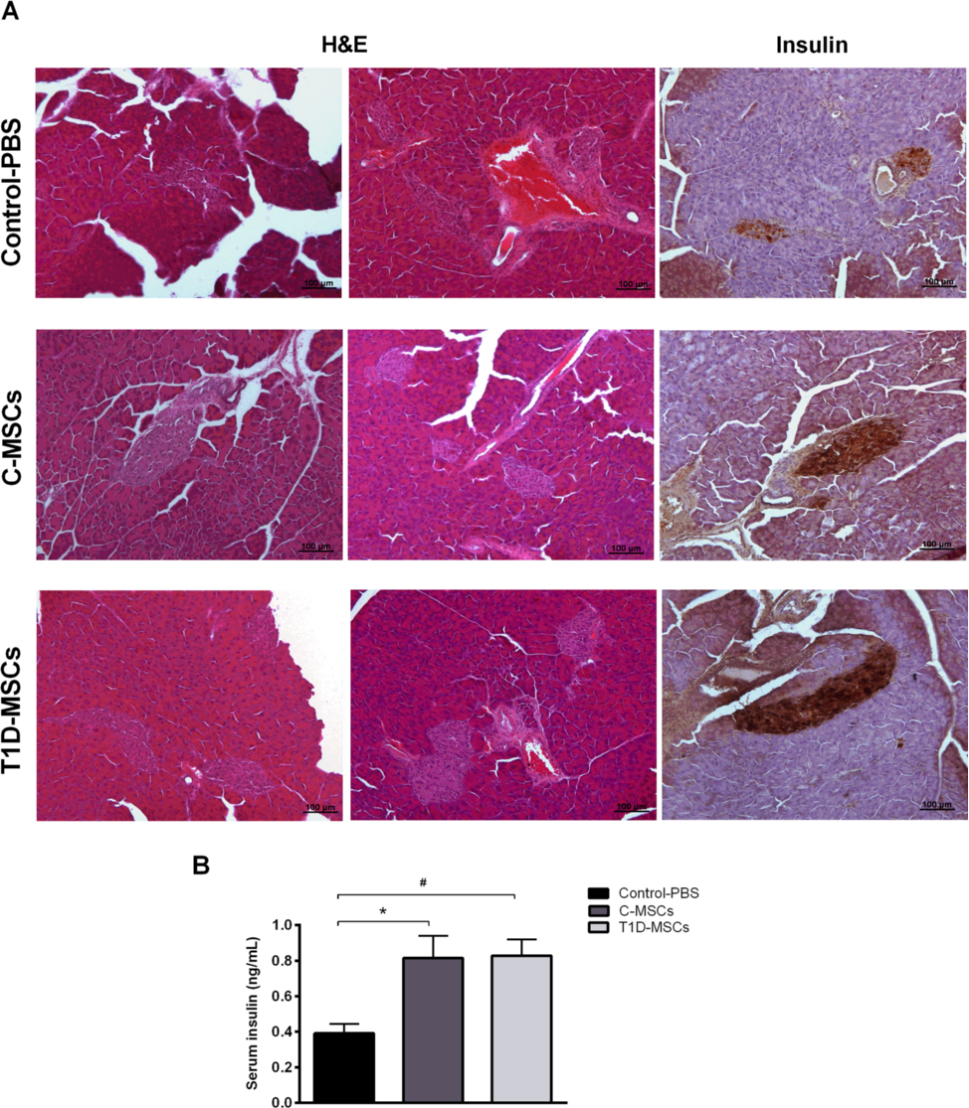
T1D-MSC transplantation reduces insulitis and augments insulin production by pancreatic β cells in diabetic-treated mice. **a** Pancreata from Control-PBS-treated, C-MSC-treated, and T1D-MSC-treated mice were collected 35 days after the treatments. Islet morphology was evaluated by H & E staining and the in situ insulin content was detected by immunohistochemistry analysis. Representative H & E or insulin-stained islets from the Control-PBS group (*upper panel*), C-MSC-treated group (*middle panel*), and T1D-MSC-treated group (*lower panel*) are shown. Original magnification: 100×. **b** Blood samples were collected on day 35 and circulating-insulin levels were determined by ELISA. Bars represent mean ± SD. *P <0.05 (Control-PBS × C-MSCs); ^#^ *P* <0.05 (Control-PBS × T1D-MSCs). *C-MSCs* mesenchymal stromal cells from bone marrow of healthy individuals, *H & E* hematoxylin and eosin, *PBS* phosphate-buffered saline, *T1D-MSCs* mesenchymal stromal cells from bone marrow of newly diagnosed T1D patients

Pancreatic islets of PBS-treated diabetic mice presented an inflammatory process (insulitis) 35 days after administration of saline (Additional file 3: Figure S2). On the other hand, C-MSC-treated and T1D-MSC-treated responder mice did not exhibit islet infiltration in the same period. Moreover, pancreatic islets from MSC-treated responder mice were larger in size than those observed in the control group (Fig. 5a).

We also investigated whether MSC therapy could preserve or regenerate β cells by evaluating in situ insulin expression in pancreatic islets. Pancreatic islets of T1D-MSC-treated mice showed higher in situ insulin expression compared with the control group, and similar to the C-MSC-treated group (Fig. 5a). The presence of Ki-67-positive cells, reflecting pancreatic cells under proliferation, was similar in all experimental groups (Additional file 4: Figure S3).

The concentration of serum insulin was higher in T1D-MSC-treated responder mice (0.82 ± 0.09 ng/ml, *P* <0.007) and C-MSC-treated responder mice (0.81 ± 0.12 ng/ml, *P* <0.02) compared with the control group levels (0.39 ± 0.05 ng/ml) 35 days after MSCs or PBS administration (Fig. 5b).

### T1D-MSC treatment does not affect the frequency of CD4^+^CD25^+^Foxp3^+^ Treg cells in the spleen and lymph nodes

**Fig. 6:**
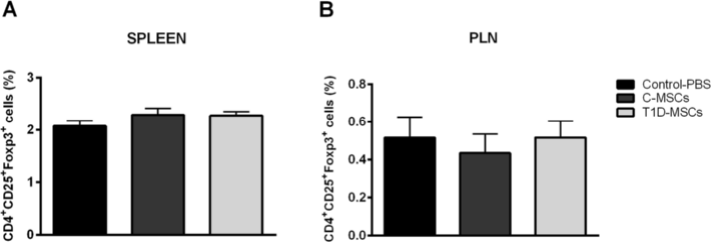
Treatment with T1D-MSCs does not affect the frequency of Treg cells in spleens and PLNs of diabetic mice. Frequency of regulatory CD4^+^CD25^+^Foxp3^+^ T (Treg) cells was analyzed by flow cytometry in cell suspensions obtained from the **a** spleen and **b** PLN from T1D-MSC-treated, C-MSC-treated, and PBS-treated mice. Cells were stained for surface markers CD4 and CD25, and subsequently for the transcription factor Foxp3. Bars represent mean ± standard error of the mean. *C-MSCs* mesenchymal stromal cells from bone marrow of healthy individuals, *PBS* phosphate-buffered saline, *PLN* pancreatic draining lymph nodes, *T1D-MSCs* mesenchymal stromal cells from bone marrow of newly diagnosed T1D patients

To evaluate whether T1D-MSC or C-MSC treatment was associated with the induction/expansion of Treg cells, we analyzed the frequency of CD4^+^CD25^+^Foxp3^+^ T cells in the spleen and PLN 35 days after PBS/MSC administration. Frequencies of CD4^+^CD25^+^Foxp3^+^ T cells in the spleen and PLN were similar in T1D-MSCs (2.27 ± 0.18 % and 0.51 ± 0.21 %), C-MSCs (2.28 ± 0.30 % and 0.43 ± 0.24 %), and control (2.07 ± 0.19 % and 0.51 ± 0.21 %) groups, respectively (Fig. 6).

### T1D-MSC treatment modulates cytokine levels in the pancreatic microenvironment

**Fig. 7:**
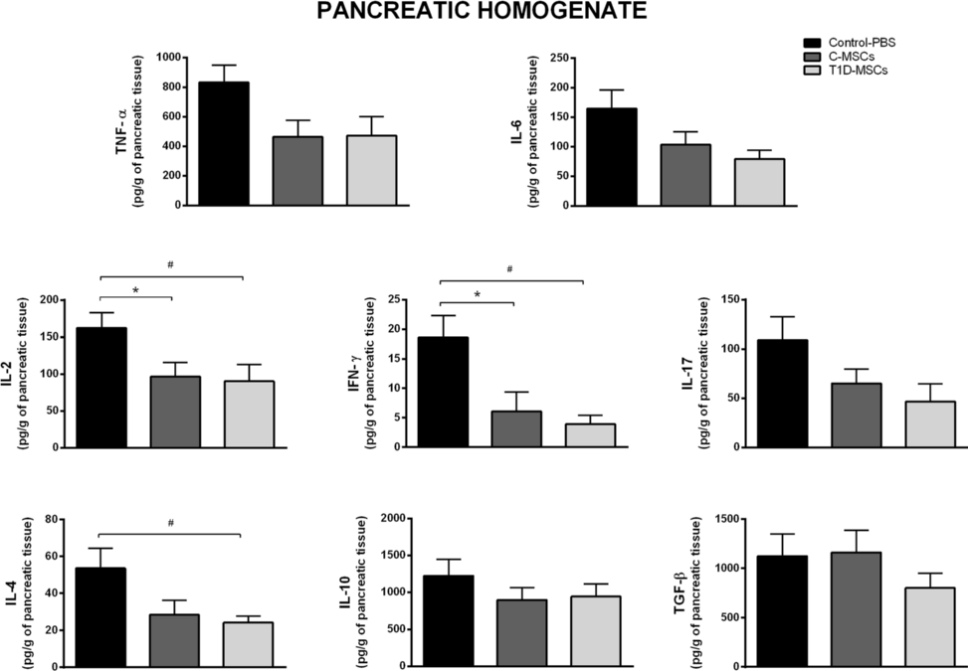
Intrasplenic T1D-MSC administration modulates proinflammatory cytokines in the pancreatic tissue of STZ-induced diabetic mice. Pancreata were obtained from Control and MSC-treated groups 35 days after treatment. The samples were weighed and homogenized in the presence of proteases inhibitor. Levels of IL-2, IL-4, IL-17, IL-6, IFNγ, TNFα, and IL-10 were measured by cytokine beads array (CBA) method. The TGF-β level was quantified by ELISA. Cytokine concentrations are represented by picograms of protein per gram of pancreatic tissue. Bars represent mean ± SD. *P <0.05 (Control-PBS × C-MSCs); ^#^ *P* <0.05 (Control-PBS × T1D-MSCs). *C-MSCs* mesenchymal stromal cells from bone marrow of healthy individuals, *IFN* interferon, *IL* interleukin, *PBS* phosphate-buffered saline, *T1D-MSCs* mesenchymal stromal cells from bone marrow of newly diagnosed T1D patients, *TGF-β* transforming growth factor beta, *TNF*α tumor necrosis factor alpha

The levels of proinflammatory (IL-2, IL-6, IFNγ, TNFα, IL-17) and anti-inflammatory (IL-4, IL-10) cytokines were determined in the serum and pancreatic tissue homogenate. No significant differences were observed in serum cytokine levels of the different experimental groups (Additional file 5: Figure S4). Significant reduction of IL-2 and IFNγ levels was observed in the pancreas of MSC-treated responder mice (T1D-MSCs group: 90.20 ± 55.86 pg/g and 3.92 ± 3.60 pg/g; C-MSCs group: 96.72 ± 46.36 pg/g and 6.09 ± 5.25 pg/g; control group: 161.96 ± 47.22 pg/g and 18.60 ± 8.22 pg/g; *P* <0.05). The levels of pancreatic IL-4 were diminished in the T1D-MSC-treated group (24.24 ± 8.58 pg/g) compared with the control group (53.61 ± 24.05 pg/g, *P* = 0.02). Levels of IL-6, TNFα, and IL-17 were slightly decreased in the pancreas of C-MSC-treated and T1D-MSC-treated responder mice compared with the control group; however, these differences were not significant (*P* >0.05; Fig. 7).

## Discussion

MSCs have been considered to be a promising therapeutic approach for inflammatory diseases and AID, particularly because of their immunomodulatory properties. The ability of MSCs to suppress the immune response suggests a possible role of these cells to promote tolerance in AID, and supports their application in the treatment of T1D. Satisfactory results of experimental studies [20, 31–35] have encouraged the use of MSCs in several T1D clinical trials worldwide [22]. However, researchers and physicians still debate the best source of cells: allogeneic versus autologous MSCs. In an autologous transplantation setting, the cells are not rejected by the host immune system, additional immunosuppressive treatment is dispensable, and transfer of donor-derived infections is unlikely. However, some pathological conditions may affect MSC viability and functions, limiting their use for autologous transplantation. In fact, MSCs isolated from patients with AID presented defects in critical functions. Impaired hematopoietic support, premature loss of telomere length, and increased production of TNFα have been reported in MSCs from patients with RA [28, 36, 37]. Similarly, MSCs isolated from MS [29], systemic lupus erythematosus (SLE) [38], and ITP [30] patients present defective immunomodulatory properties. Whether phenotypical and functional properties of MSCs derived from patients with T1D are normal or somehow defective has not yet been established. Since the autoimmune process and diabetic microenvironment may change the biology of MSCs, it becomes important to investigate whether these cells are suitable for autologous transplantation. Therefore, in this study we analyzed MSCs isolated from the bone marrow of newly diagnosed T1D patients and compared them with their healthy counterparts.

T1D-MSCs exhibited typical morphology and similar cell size compared with C-MSCs. Similarly, MSCs obtained from MS [26, 39], RA [37], SS [27, 40, 41], and Crohn’s disease [42] patients did not present morphological alterations compared with healthy counterparts. On the other hand, MSCs isolated from patients with ITP [30] and SLE [43] showed atypical morphology.

In our study, T1D-MSCs and C-MSCs presented typical expression of MSC surface markers and both cell types were able to differentiate towards an adipocyte lineage. Reports from the literature also describe that MSCs from patients with MS [26, 39], SS [27, 40, 41], SLE [43], or ITP [30] present similar immunophenotypic profiles and in vitro differentiation capacity compared with C-MSCs.

The immunomodulatory mechanisms of MSCs have been explored extensively. A wide range of soluble factors has been involved in MSC immunomodulatory function, including HGF, prostaglandin E_2_, TGF-β1, IDO, nitric oxide (NO), IL-10, heme oxygenase-1, and HLA-G [16, 44, 45]. MSCs develop their immunosuppressive functions after being exposed to the inflammatory environment and this “licensing” step is provided by molecules of acute phase inflammation, such as IFNγ and TNFα, or TLR ligands [25]. In our microarray analysis, the expression of immunomodulatory genes (*PDL1*, *NOS2*, *IL10*, *PTGES*, *TGFB1*, *PDL2*, *HLAG*, and *TGS6*) and licensing-related genes (*IFNGR2*, *TNFR1*, *IFNGR1*, *TNFR2*, *TLR4*, and *TLR3*) was similar in T1D-MSCs and C-MSCs. However, expression of the *HGF* gene was downregulated in T1D-MSCs. In a previous publication from our group [29], microarray analysis of MSCs isolated from MS patients and healthy controls revealed 618 differentially expressed genes, some of them related to the impaired immunosuppressive capacity of MSCs isolated from MS patients. Interestingly, downregulation of *HGF* and *TGFB1* genes and modulation of HGF and FGF signaling pathways were reported.

In our present study, both patient and healthy donor MSCs similarly inhibited allogeneic lymphocyte proliferation in a dose-dependent manner. Likewise, MSCs isolated from patients with juvenile idiopathic arthritis [46], Crohn’s disease [42], SS [27], and SLE [43, 47] showed preserved immunosuppressive capacity. Conversely, MSCs from MS [29] and ITP [30] patients exhibited less in vitro T-cell antiproliferative activity when compared with C-MSCs. Taking into account our in silico and in vitro analyses, we suggest that T1D-MSCs have preserved immunomodulatory function.

The therapeutic potential of T1D-MSCs to modulate disease progression was tested in the STZ-induced diabetes model. MSCs were injected in mice 20 days after diabetes induction, in the chronic phase of disease progression characterized by hyperglycemia, massive β-cell destruction, and α-cell expansion with disruption of the pancreatic islet architecture [48]. Different MSC delivery routes were tested previously by our research group and the intrasplenic route was the more effective to reverse hyperglycemia in diabetic-treated mice [49]. The intrasplenic administration of MSCs effectively reversed diabetes in 67 % of treated mice. Both T1D-MSC and C-MSC applications equivalently contributed to increase β-cell mass, insulin production, and glucose tolerance. We can therefore infer that T1D-MSCs do not present functional abnormalities. Accordingly, Dong et al. showed that MSCs isolated from diabetic rats decreased blood glucose levels and prevented body weight loss when transplanted into diabetic animals. The authors suggest that diabetes does not influence MSC properties, supporting the use of autologous MSCs in the treatment of T1D patients [50]. On the contrary, Fiorina et al. reported that MSCs isolated from nonobese diabetic (NOD) mice were unable to delay the onset of diabetes when administered to prediabetic NOD mice and, furthermore, did not reverse hyperglycemia in mice with already established diabetes. The authors then suggested that transplantation of MSCs derived from nondiabetic donors, rather than autologous MSCs, would be the best option for the treatment of T1D [21].

Studies have demonstrated the benefic role of MSCs on in vivo and in vitro induction/proliferation of Treg cells [51–53]. In our study, intrasplenic administration of T1D-MSCs or C-MSCs did not affect the frequency of CD4^+^CD25^+^Foxp3^+^ Treg cells in spleens and PLNs of diabetic mice 35 days after MSC therapy. Fiorina et al. [21] also did not observe significant modifications in the frequency of Treg CD4^+^CD25^+^Foxp3^+^ cells in spleens and PLN of NOD-treated mice 28 days after MSC administration. However, opposing results were reported by Madec et al. [20], who observed an increase in CD4^+^Foxp3^+^ Treg cells 5 days after administration of MSCs in diabetic NOD mice. Increased frequency of Treg cells was also observed in STZ-induced diabetic mice 7 days after MSC transplantation [31, 33]. Additionally, Jurewicz et al. reported increased levels of the regulatory cytokine IL-10 in NOD mice 7 days after MSC injection. However, this elevation was not detected on days 14 and 21 after cell therapy [32]. Our analyses were performed 35 days after MSC administration, which may represent too long a period of time to detect alterations in Treg cell frequency. We therefore believe that further experiments should be performed earlier after cell transplantation, to characterize immediate immune alterations promoted by injected MSCs. Our results thus indicate that the beneficial effects promoted by administration of T1D-MSCs or C-MSCs are not related to late expansion of Treg cells and that other mechanisms may be responsible for the effective therapeutic response.

An inflammatory process (insulitis) was observed in the pancreatic islets of PBS-treated mice but not in those from MSC-treated mice. Additionally, levels of IL-2 and IFNγ were decreased in the pancreatic homogenate of T1D-MSC-treated and C-MSC-treated mice 35 days after MSC administration. Moreover, a marginal but not substantial decrease in levels of IL-6, TNFα, and IL-17 in the pancreatic tissue was observed after MSC injections. Accordingly, allogeneic MSC therapy decreased levels of pancreatic IFNγ in NOD-treated mice [34]. Ezquer et al. [31] also observed that MSC therapy induced a shift from a proinflammatory (IL-2, IFNγ, and TNFα) to an anti-inflammatory (IL-13) cytokine profile in diabetic mice. The decrease of proinflammatory cytokine production observed in our work corroborates these studies and may represent a possible mechanism by which MSCs prevent pancreatic β-cell death and promote reversion of hyperglycemia in the diabetic treated mice. Moreover, the constraint of pancreatic inflammation enables the preservation of residual and/or newly formed β cells with regular insulin production.

MSCs from patients with chronic AID, such as MS [29] and RA [28], showed abnormal cell characteristics and functions compared with MSCs from healthy counterparts. In these chronic cases, some endogenous alterations or previous immunomodulatory/immunosuppressive therapies may have changed the bone marrow microenvironment and affected MSC intrinsic pathways [10]. On the other hand, we studied MSCs isolated from T1D patients who were diagnosed in the previous 6 weeks, corresponding to early stages after clinically overt disease. Recently, Carlsson et al. treated recently diagnosed T1D patients (<3 weeks before enrollment) with autologous MSCs. Autologous MSC transplantation was safe and was able to preserve or even increase C-peptide levels in T1D patients [23]. Although limited by the small number of treated patients and short follow-up, the results from Carlsson et al. [23] reinforce our data. We can suggest that MSCs from recently diagnosed T1D individuals have their biological and functional properties preserved, since there is no prolonged exposure to harmful inflammatory and metabolic diabetic conditions. Further studies using MSCs from chronic T1D patients will be necessary to evaluate the influence of long-term diabetic environment on bone marrow MSC biological properties.

## Conclusions

Our study provided for the first time a detailed characterization of MSCs isolated from the bone marrow of newly diagnosed T1D patients. Compared with their healthy counterparts, T1D-MSCs showed similar morphology, immunophenotypic characteristics, adipocyte differentiation potential, expression of immunomodulatory genes, and in vitro immunosuppressive capacity. When administered to diabetic mice, T1D-MSCs and C-MSCs similarly and successfully reversed hyperglycemia, improved β-cell mass, increased insulin production, and modulated pancreatic cytokine production. The present results provide support for the use of autologous MSCs to treat patients with recently diagnosed T1D.

## Additional files

Additional file 1:
**Presents the supplementary methods.** (DOCX 17 kb) Additional file 2: Figure S1. Showing the experimental design. Diabetes was induced in C57BL/6 male mice after daily intraperitoneal injections of 40 mg/kg STZ for 5 consecutive days. Twenty days after diabetes induction, mice were randomly divided into three experimental groups: Control-PBS group (diabetic mice treated with PBS; *n* = 6), C-MSCs group (diabetic mice treated with 1 × 10^6^ MSCs isolated from healthy individuals; *n* = 9), and T1D-MSCs group (diabetic mice treated with 1 × 10^6^ MSCs isolated from newly diagnosed T1D patients; *n* = 9). Nonfasting glucose blood levels were frequently determined. Thirty-five days after PBS/MSC administration, mice were sacrificed and different tissue samples were collected and analyzed. (TIFF 381 kb) Additional file 3: Figure S2. Showing the inflammatory process (insulitis) into the pancreatic islets of PBS-treated diabetic mice. The pancreas of diabetic mice was collected 35 days after PBS administration. Pancreatic tissue section was analyzed by H & E staining. Insulitis is indicated by the *arrows*. Original magnification: 100 × . (TIFF 8375 kb) Additional file 4: Figure S3. Showing that C-MSC or T1D-MSC transplantation does not change Ki-67 expression in pancreatic islet cells of diabetic mice. Pancreata from the Control group, C-MSC-treated mice, and T1D-MSC-treated mice were collected 35 days after the treatment. Pancreatic tissue sections were analyzed by immunohistochemistry with anti-Ki-67 antibody to evaluated proliferating pancreatic islet cells. Representative images of pancreatic islets from Control-PBS group (*left*), C-MSC-treated group (*middle*) and T1D-MSC-treated group (*right*) are shown. Magnification: 200 × . (TIFF 790 kb) Additional file 5: Figure S4. Showing the profile of serum proinflammatory and anti-inflammatory cytokines does not change after intrasplenic T1D-MSC administration. Blood samples were obtained from Control and MSC-treated groups 35 days after treatment. Samples were centrifuged and serum collected. Levels of IL-2, IL-4, IL-17, IL-6, IFNγ, TNFα, and IL-10 were measured by cytokine beads array (CBA) method. Cytokine concentrations are represented as picograms of protein per milliliter of serum. Bars represent mean ± SD. (TIFF 57 kb)

## Abbreviations

AID: Autoimmune diseases
AUC: Area under the glycemia curve
CBA: Cytometric bead array
CFSE: Carboxyfluorescein diacetate succinimidyl ester
C-MSC: Multipotent mesenchymal stromal cell from bone marrow of healthy individuals (control)
EDTA: Ethylenediamine tetraacetic acid
FBS: Fetal bovine serum
GAD: Glutamic acid decarboxylase
GTT: Glucose tolerance test
H & E: Hematoxylin and eosin
HGF: Hepatic growth factor
HLA: Human leukocyte antigen
IDO: Indoleamine 2,3-dioxygenase
IFNγ: Interferon gamma
IFNGR: Interferon gamma receptor
IL: interleukin
ITP: Immune trombocytopenic purpura
αMEM: Alpha minimum essential medium
MHC: Major histocompatibility complex
MS: Multiple sclerosis
MSC: Multipotent mesenchymal stromal cell
NO: Nitric oxide
NOD: Nonobese diabetic
NOS2: Nitric oxide synthase 2
PBMC: Peripheral blood mononuclear cell
PBS: Phosphate-buffered saline
PDL: Programmed death ligand
PLN: Pancreatic draining lymph nodes
PTGES: Prostaglandin E synthase
RA: Rheumatoid arthritis
SD: Standard deviation
SLE: Systemic lupus erythematosus
SS: Systemic sclerosis
STZ: Streptozotocin
T1D: Type 1 diabetes mellitus
T1D-MSC: Multipotent mesenchymal stromal cell isolated from bone marrow of newly diagnosed T1D patients
TGF-β: Transforming growth factor beta
TGS6: Tumor necrosis factor-inducible gene 6 protein
TLR: Toll-like receptor
TNFR: Tumor necrosis factor receptor
Treg: Regulatory T
