# Supplementary material for: Multipotent mesenchymal stromal cells from patients with newly diagnosed type 1 diabetes mellitus exhibit preserved in vitro and in vivo immunomodulatory properties
Source: Stem Cell Res Ther. 2016 Jan 18;7:14. doi: 10.1186/s13287-015-0261-4 (PMC4861132; doi:10.1186/s13287-015-0261-4)
Supplement: Additional file 1: — Presents the supplementary methods. (DOCX 17 kb) [file 13287_2015_261_MOESM1_ESM.docx]

**Additional File 1: Supplementary Methods**

***MSCs morphology***

MSCs were expanded from bone marrow samples of healthy donors (C-MSCs) or T1D patients (T1D-MSCs), cultured until third passage and observed under inverted Axiovert 40 CFL (Carl Zeiss, Goetingen, Germany) microscope. Subsequently, MSCs were stained with Leishman and observed under light microscope (Nikon TS100, China). MSCs size (diameter) was determined using Vi-Cell XR Coulter Counter equipment (Beckman Coulter, Brea, CA, USA).

***MSCs immunophenotypic profile***

C-MSCs and T1D-MSCs at third passage were incubated with the following monoclonal antibodies anti-: CD45, CD14, CD29, CD51/61, CD44, CD90, CD105, CD54, CD73, CD106, CD34, HLA-CLASS I (HLA-A/B/C), HLA- CLASS II (HLA-DRB1), CD166, CD49e, CD13, STRO-1 and KDR (Becton‐Dickinson/BD, San Jose, CA, USA) for 30 minutes at room temperature in the dark. Flow cytometry analysis was performed using FACSCalibur^TM^ (BD) equipment and 20,000 cells were acquired and analyzed by the CellQuest Pro software (BD).

***Adipocyte differentiation potential***

*In vitro* adipogenic differentiation of C-MSCs and T1D-MSCs was induced using α-MEM medium supplemented with 15% FBS (Gibco), 100 mM dexamethasone (Prodome, Campinas, SP, Brazil), 10 μg/mL insulin (Sigma‐Aldrich, Saint Louis, MO, USA) and 100 μM indomethacin (Sigma‐Aldrich). For negative controls, MSCs were cultured with α-MEM medium supplemented with 15% FBS. Culture medium was changed every three days and the cells were maintained in culture for 21 days. MSCs were then fixed with ethanol and stained with Sudan II-Scarlet and Harris hematoxylin. The presence of lipid vacuoles into the MSCs was observed under light microscopy. For each group, one hundred of differentiated adipocytes were randomly chosen and their size (area) was determined using the ImageJ computerized system (National Institutes of Health, ML) for morphometry.
