# Supplementary figures and images for: Multipotent mesenchymal stromal cells from patients with newly diagnosed type 1 diabetes mellitus exhibit preserved in vitro and in vivo immunomodulatory properties
Source: Stem Cell Res Ther. 2016 Jan 18;7:14. doi: 10.1186/s13287-015-0261-4 (PMC4861132; doi:10.1186/s13287-015-0261-4)

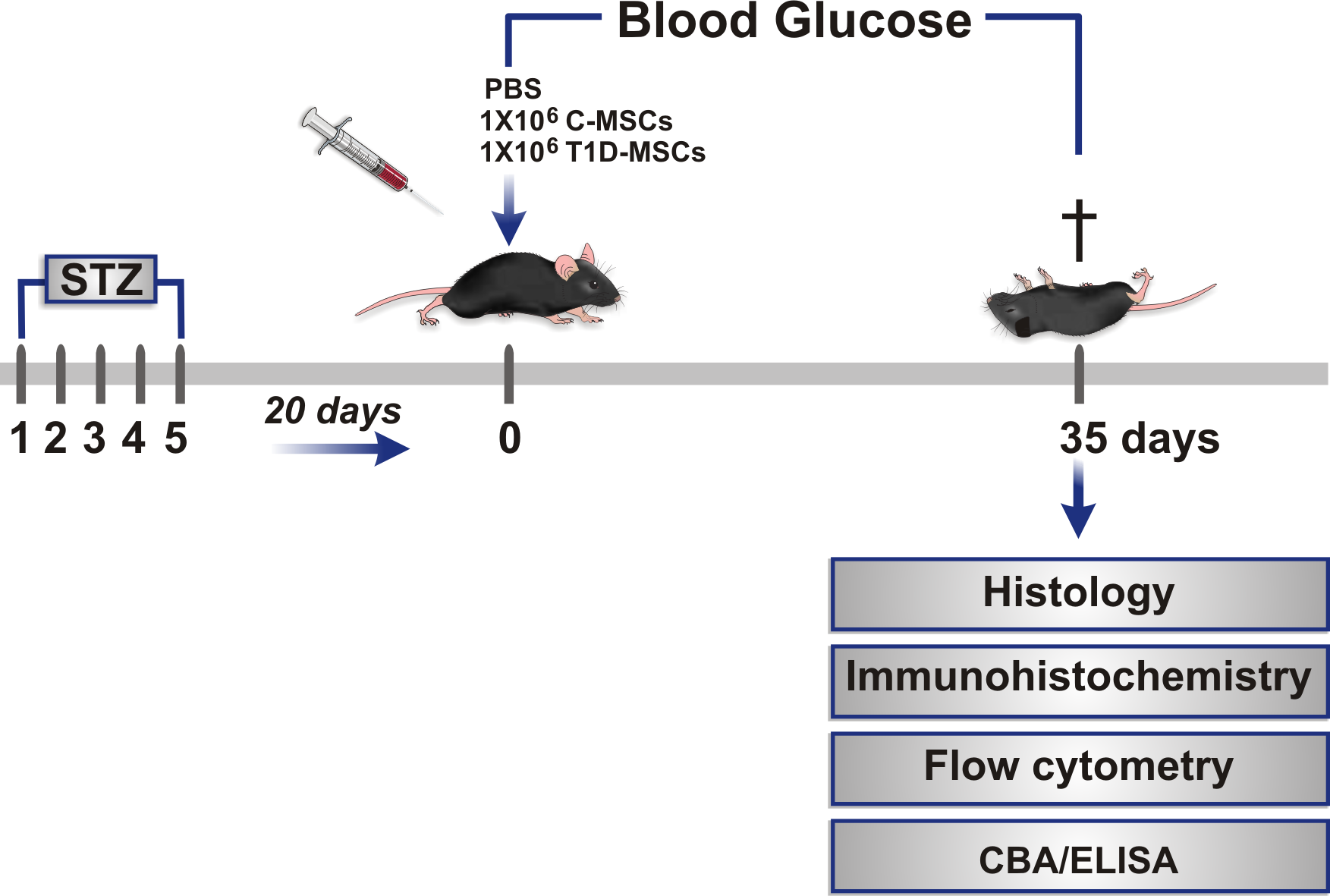

Supplement: Additional file 2: Figure S1. — Showing the experimental design. Diabetes was induced in C57BL/6 male mice after daily intraperitoneal injections of 40 mg/kg STZ for 5 consecutive days. Twenty days after diabetes induction, mice were randomly divided into three experimental groups: Control-PBS group (diabetic mice treated with PBS; n = 6), C-MSCs group (diabetic mice treated with 1 × 10^6^ MSCs isolated from healthy individuals; n = 9), and T1D-MSCs group (diabetic mice treated with 1 × 10^6^ MSCs isolated from newly diagnosed T1D patients; n = 9). Nonfasting glucose blood levels were frequently determined. Thirty-five days after PBS/MSC administration, mice were sacrificed and different tissue samples were collected and analyzed. (TIFF 381 kb) [file 13287_2015_261_MOESM2_ESM.tiff]

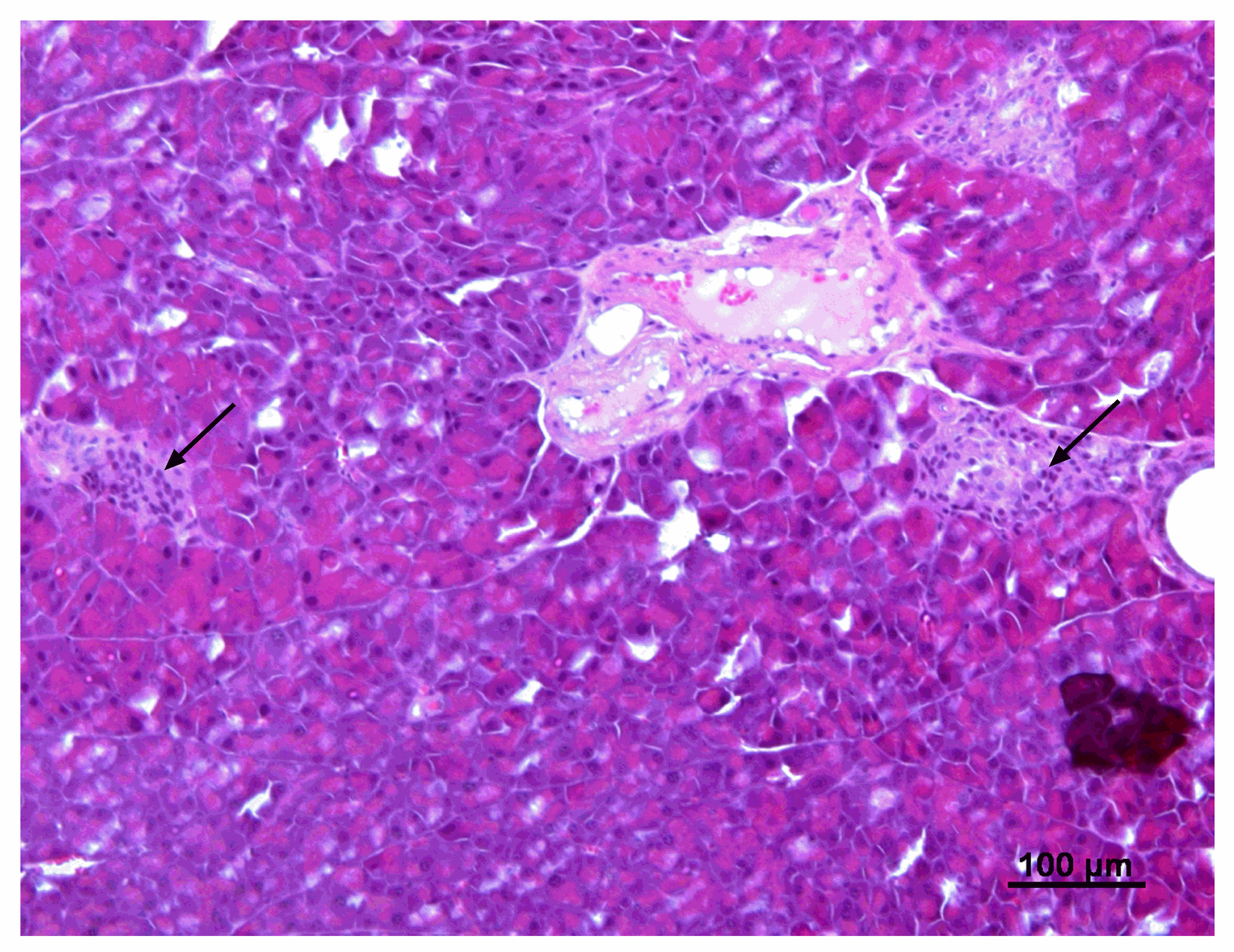

Supplement: Additional file 3: Figure S2. — Showing the inflammatory process (insulitis) into the pancreatic islets of PBS-treated diabetic mice. The pancreas of diabetic mice was collected 35 days after PBS administration. Pancreatic tissue section was analyzed by H & E staining. Insulitis is indicated by the arrows. Original magnification: 100 × . (TIFF 8375 kb) [file 13287_2015_261_MOESM3_ESM.tiff]
